# Angiogenesis and bleeding disorders in FNAIT

**DOI:** 10.18632/oncotarget.4757

**Published:** 2015-07-03

**Authors:** Issaka Yougbare, Darko Zdravic, Heyu Ni

**Affiliations:** Toronto Platelet Immunobiology Group, Canadian Blood Services and Department of Laboratory Medicine and Pathobiology, Keenan Research Centre for Biomedical Science, St. Michael's Hospital, University of Toronto, Toronto, ON, Canada

Angiogenesis is a physiological process essential for embryo/fetal growth, wound healing, and repair of the myocardium after myocardial infarction. Angiogenesis is also implicated in pathological processes such as retinopathy and tumor growth. Anti-angiogenic agents have been demonstrated to have promising therapeutic potential for inhibiting tumour growth. Earlier studies suggested that β3 integrin (i.e. αVβ3 that is formed by β3 subunit and αV subunit) expressed on angiogenic endothelial cells (ECs), is required for angiogenesis [1]; however, subsequent studies demonstrated that β3 integrin deficiency (β3^−/−^) did not prevent embryo/fetal growth and interestingly, enhanced pathological angiogenesis was observed in β3^−/−^ mice [2]. It is therefore important to further study the roles of β3 integrin in angiogenesis, fetal development and related diseases.

Fetal and neonatal alloimmune thrombocytopenia (FNAIT) is a life-threatening bleeding disorder that occurs when maternal alloantibodies cross the placenta and target paternally derived antigens, especially GPIIbIIIa (integrin αIIbβ3) and GPIbα, on fetal/neonatal platelets. In contrast to αVβ3, integrin αIIbβ3 is composed of a β3 subunit and αIIb subunit, which are almost exclusively expressed on platelets and megakaryocytes. Half of the polymorphisms known to cause FNAIT are located on the β3 subunit. Approximately 80-90% of reported FNAIT cases are caused by antibodies targeting Human-Platelet-Antigen-1a (HPA-1a) on β3 subunit. Antibodies targeting the HPA-2a on GPIbα have also been reported. Intracranial hemorrhage (ICH), which occurs in 10-20% of affected fetuses/neonates, is a major clinical complication of FNAIT, leading to neurological impairment and death. Unfortunately, the mechanism responsible for ICH, has only been inferred but not adequately explored.

Thrombocytopenia was considered to be the cause of bleeding in FNAIT. Interestingly, mice deficient in transcription factor NF-E2, which lack circulating platelets, did not develop significant bleeding disorders in utero. More strikingly, the murine fetuses with combined deficiencies in NF-E2 and fibrinogen genes exhibited normal embryonic development and were morphologically indistinguishable from their wild-type control counterparts at 18.5 days postcoitum [3]. Therefore, it is likely that neither thrombocytopenia nor blood coagulation are crucial for the development of ICH in FNAIT. Furthermore, several animal models have demonstrated that impairment of angiogenesis may contribute to bleeding in fetuses, particularly in their brains, since the brain is one of the major sites of angiogenesis during fetal development. However, whether ICH in FNAIT is caused by impairment of angiogenesis induced by maternal anti-platelet antibodies, is unclear. This is particularly interesting since the roles of β3 integrin in angiogenesis are still controversial.

In a recent publication [4], we reported both active and passive murine models of FNAIT using β3−/− and GPIbα−/− mice, in which gene deficient female mice were bred with wild-type males to mimic human FNAIT. We found that both maternal anti-β3 and anti-GPIbα antibodies caused severe thrombocytopenia, however, ICH was only observed in anti-β3-mediated FNAIT fetuses/neonates (active model), and only anti-β3 sera-injected neonates (passive model) developed ICH. Moreover, we found that anti-β3 but not anti-GPIbα antibodies induced EC apoptosis, decreased the vessel density in the brains, and impaired the retinal vascular development. The anti-β3 antibodies also decreased the expression of several pro-angiogenic factors, increased the expression of anti-angiogenic factors, and down-regulated Akt signalling. The antibodies also inhibited proliferation and network formation of human umbilical vein endothelial cells (HUVECs) *in vitro*. Importantly, anti-HPA-1a IgG purified from mothers who gave birth to neonates with FNAIT, also showed similar effects on HUVECs *in vitro*. This study suggests that anti-platelet β3 antibodies also cross-react with αVβ3 on ECs, thereby impairing angiogenesis and contributing to ICH. The impairment of retinal vascular development may explain why some FNAIT untreated siblings suffered visual impairment in adulthood. Our study may also provide insights into the mechanisms of intrauterine growth restriction (IUGR) in FNAIT as well as the possible pathogenic role of αVβ3 integrin in tumorigenesis.

Clinical management of such devastating bleeding diatheses in fetuses/neonate remains a formidable challenge. Since our findings indicate that impairment of angiogenesis but not thrombocytopenia may be the major cause of ICH, invasive fetal platelet transfusion may not be an effective prenatal therapy. Our data demonstrated that antenatal administration of intravenous immunoglobulin (IVIG) decreased EC apoptosis, rescued brain and retinal vessel development, increased platelet counts, and ameliorated ICH, suggesting that IVIG may be an effective therapy [4]. In addition, our previous studies demonstrated that fetal (but not maternal) neonatal Fc receptor (FcRn) is required for IgG trans-placental transport [6-7]. Whether anti-FcRn is a more effective and economical therapy for FNAIT requires further clinical studies.

Aside from its expression on platelets and ECs, β3 integrin is also expressed on highly invasive trophoblasts, which are essential for normal placental vascular remodeling and development. Therefore, further studies on the effects of anti-β3 antibodies on trophoblast proliferation, differentiation, migration, and invasion, may provide insights into the mechanism of IUGR and miscarriage in FNAIT, as well as aid in the development of new therapies to manage these disorders.

## References

[1] Brooks P.C. (1994). Cell.

[2] Reynolds L.E. (2002). Nat Med.

[3] Palumbo J.S. (2004). J Thromb Haemost.

[4] Yougbaré I. (2015). J Clin Invest.

[5] Ni H. (2006). Blood.

[6] Chen P. (2010). Blood.

[7] Li C. (2011). J Clin Invest.

